# 3D Object Detection Using Multiple-Frame Proposal Features Fusion

**DOI:** 10.3390/s23229162

**Published:** 2023-11-14

**Authors:** Minyuan Huang, Henry Leung, Ming Hou

**Affiliations:** 1 Department of Electrical and Software Engineering, University of Calgary, Calgary, AB T2N 1N4, Canada; leungh@ucalgary.ca; 2 Defence Research and Development Canada (DRDC), Toronto, ON B3K 5X5, Canada; ming.hou@forces.gc.ca

**Keywords:** autonomous driving, 3D object detection, multiple frame point clouds, feature and data fusion

## Abstract

Object detection is important in many applications, such as autonomous driving. While 2D images lack depth information and are sensitive to environmental conditions, 3D point clouds can provide accurate depth information and a more descriptive environment. However, sparsity is always a challenge in single-frame point cloud object detection. This paper introduces a two-stage proposal-based feature fusion method for object detection using multiple frames. The proposed method, called proposal features fusion (PFF), utilizes a cosine-similarity approach to associate proposals from multiple frames and employs an attention weighted fusion (AWF) module to merge features from these proposals. It allows for feature fusion specific to individual objects and offers lower computational complexity while achieving higher precision. The experimental results on the nuScenes dataset demonstrate the effectiveness of our approach, achieving an mAP of 46.7%, which is 1.3% higher than the state-of-the-art 3D object detection method.

## 1. Introduction

With the development of deep learning, computer vision has witnessed a rise in several areas, such as image object detection [1,2] and image segmentation [3]. Object detection using cameras has found widespread application in various fields. RGB images offer the advantage of low acquisition cost, high image resolution, and the inclusion of semantic information such as object color and texture. However, they are susceptible to environmental influences, such as weather and lighting conditions, and lack depth information. With the development of remote sensing technology, LiDAR, a remote sensing instrument, has been widely used by researchers to capture data; for example, 3D point clouds can be acquired from LiDAR sensors. These 3D point cloud data provide accurate geometric information, which is widely used in tracking reconstruction areas and has also been considered in the detection topic.

The 3D point cloud collected by LiDAR includes spatial coordinates (X, Y, Z) and reflection intensity, offering high detection accuracy and providing precise scene information for 3D object detection. Standard outdoor datasets include the Kitti, nuScenes, and Waymo.

**Figure 1:**
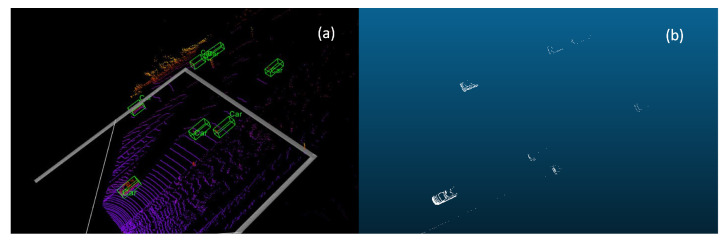
Ambiguous objects in point cloud data. (**a**) the whole point cloud (**b**) the points for ground truth objects. Green boxes refer to the ground truth box in (**a**).

Although point cloud data offer various advantages, they have some limitations. When objects are located at a far distance or heavily occluded, point cloud data would be sparse, leading to unclear object representations and making detection difficult. Figure 1 illustrates this problem by demonstrating ambiguous objects in point cloud data. The input point cloud data are visualized in (a), with green boxes representing the 3D ground truth box projection, while (b) shows the points representing objects in the input point cloud. The smallest ground truth object contains only 20 points. It can be observed that points in distant regions are pretty sparse, and their shapes are difficult to recognize. Although increasing the number of LiDAR scan lines can alleviate this problem, it would significantly raise the price cost of hardware. Velodyne 64 costs USD 80,000 and can emit 64-beam lasers, while Velodyne 32 only costs USD 20,000; however, only 32-beam lasers can be emitted, which leads to a severe sparsity problem.

**Figure 2:**
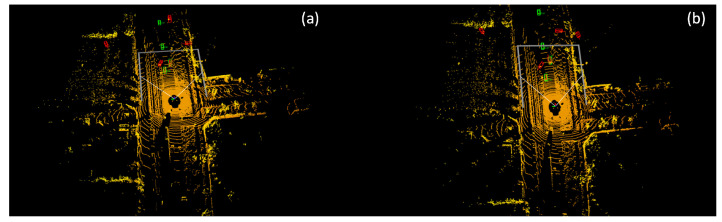
Failure examples of single-frame detector on multiple frame dataset using SECOND [4]. Green boxes refer to the ground truth boxes, and red boxes refer to the detection boxes.

As shown in Figure 2, green boxes refer to the ground truth boxes, and red boxes are prediction boxes. Single-frame detectors failed to detect the objects and generated lots of false predictions in distant areas. Figure 2a,b are two adjacent frames. As can be seen, the failure to detect objects in the previous frame is repeated in the following frame. In both cases, the detector failed to detect the two objects at the top of the point cloud while having multiple false positives.

**Figure 3:**
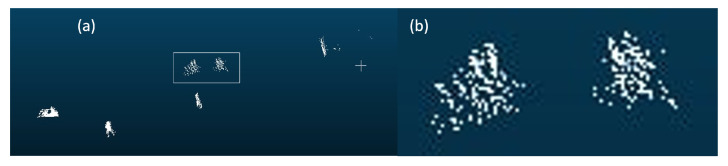
Alignment of two consecutive frames of point clouds using ICP algorithm. (**a**) represents the point cloud concatenated by two consecutive point clouds using the ICP [7] method. And (**b**) enlarges the white box to display the shadow caused by misalignment.

Using multiple-frame point clouds can effectively compensate for information. Multiple-frame point clouds, also called spatio-temporal data, can be used in several fields. For example, with spatio-temporal data, 4D dynamic scenes can be reconstructed [5,6]. In the detection field, using multiple-frame point clouds may alleviate the sparsity problem in 3D object detection. Although the above example shows the detection failed in two consecutive frames, they perform detection independently without all the data together to improve detection. With a proper fusion scheme, using multiple point cloud frames can be similar to using a denser line LiDAR. One intuitive approach is concatenating the points at input time, that is, aligning the multiple frames of point clouds into a single scene for input. Besl [7] proposed the classical iterative closest point (ICP) algorithm, which laid the foundation for point cloud registration. This method uses the sum of Euclidean distances between all points of two point clouds as the matching cost for iterative search until the matching cost is minimized. Then, the transformation matrix between the two point clouds is computed. We conducted a simple experiment using the point concatenation method, as shown in Figure 3, using ICP [7] to align the point clouds. However, this alignment approach has its drawbacks. It requires many iterations, resulting in a long computation time. Also, a suitable initial position must be provided. As we are dealing with large outdoor datasets, most objects are moving and have different velocities. The movement poses further challenges to registration, which is usually restricted to stationary objects. Despite the increased point density, shadows appear on some objects. When magnifying the point cloud representing small objects in Figure 3b, it can be observed that the alignment effect is unsatisfactory, leading to shadows on the small objects [8,9,10].

Besides registration, some approaches have been proposed for multiple-frame point clouds, such as Long Short-Term Memory (LSTM) [11] and concatenation [12,13]. However, these methods require intensive computation and suffer from the shadow problem. In this study, we propose a novel multi-frame object detection method based on fusing proposal features called proposal features fusion (PFF). The proposed method introduces an attention mechanism for feature-level fusion. Using an anchor-based detector [4], a region proposal network (RPN) is used to generate proposals for multiple frames. The cosine similarity is then utilized to associate proposal features between adjacent frames. We further propose an Attention-Weighted Fusion (AWF) module for the associated proposal features to adjust and integrate features from different frames adaptively.

We summarize our contributions as follows:

- A feature-level fusion method is proposed by fusing the extracted features from proposals of previous frames to the current frame. The feature-level fusion can improve detection performance while ensuring computational efficiency.
- We apply the attention module in feature fusion to make the model robust and flexible. The proposed Attention Weighted Fusion (AWF) module is shown to play an important role in suppressing unimportant information and enhancing key features.
- The Kitti dataset is used for the ablation study to demonstrate the effectiveness of the proposed method. The nuScenes dataset is further used to compare the performance of the proposed method with other multiple-frame point cloud methods in the literature. The comparison shows that our method outperforms the conventional multi-frame method by 6.64% mAP.

## 2. Related Work

Single-frame point cloud object detection methods can be roughly divided into two categories: point-based [14,15,16,17] and voxel-based methods [4,18,19]. Since point clouds are obtained from LiDAR scans and only contain the surface information of objects, the distances and spatial distributions between points are non-uniform. Also, point clouds exhibit sparsity and disorder. PointNet [20] and the subsequent work PointNet++ [21] use Farthest Point Sampling (FPS) to sample non-uniform points in point clouds while preserving the shape of the point cloud. These works introduced max pooling to address the disorder of point clouds. PointNet++ has been widely used as a backbone network. F-PointNet [14] employs a two-dimensional detector to generate candidate boxes and other information and then combines these 2D bounding boxes with depth information to form three-dimensional frustums. Subsequently, PointNet is used to encode the point clouds within the frustums and generate the 3D object detection results. PointRCNN utilizes PointNet++ as the backbone network and proposes a two-stage network to refine proposal boxes to achieve good detection results. Gao et al. [22] proposes a dynamic clustering algorithm by using elliptic functions as point cloud data has a non-uniform distribution. SASA [23] introduces S-FPS, an improved sampling method for small objects, to sample point clouds in the feature layer.

Another point cloud encoding method is the voxel-based method, which processes point cloud data by dividing the point cloud into 3D voxels. VoxelNet [18] proposes an end-to-end network that divides the point cloud into voxels. Then, it utilizes a voxel feature encoder (VFE) on the voxels to combine the features of individual points within each voxel and global features. A 3D CNN is then employed to predict and regress the object’s bounding box for object detection. Second [4] uses 3D sparse convolution networks [24] to accelerate 3D voxel processing. VoxelRCNN [25] proposes the utilization of voxel region of interest (ROI) pooling to optimize the features within the ROI. CenterPoint [26] utilizes a voxel-based method for point cloud encoding and introduces an anchor-free 3D box regression method for bounding boxes.

In Ref. [27], a combination method is proposed that uses both multi-scale voxel features and keypoints, Ref. [28] using both RGB and point cloud information through extracting 3D proposal boxes in the Bird’s Eye View (BEV) and project them to RGB image to obtain more features. The performance of single-frame detection is unsatisfactory due to the sparsity and occlusion in the single-frame data. With the release of the multiple-frames dataset [29,30], exploring how to utilize multiple frames has become a research topic in recent years.

In order to leverage the multiple-frame point cloud data effectively, several branches of studies have been proposed. Ref. [31] divides multiple frame point cloud studies into two branches, the data branch and the model branch. Furthermore, they classify their work into data-based approaches. Ref. [31] proposes a data augmentation method and achieves 0.7 mAP on nuScenes dataset. Ref. [32] focuses on false negative examples by using heatmap prediction to excavate hard samples and omitting the training of easy positive candidates.

Some studies [11,33] use the LSTM network to leverage spatio-temporal information in point cloud sequences. Yolo4D [33] utilizes Yolo3D [34] as the backbone network and integrates contextual information using a Recurrent Neural Network (RNN). It first employs a CNN to extract information from each frame and then feeds it into the LSTM to incorporate historical information. FaF [12] uses aligned frames as inputs and employs 3D CNN to extract features from the aligned data. However, pre-aligning multiple frames of point clouds leads to an increased processing time and computational complexity. WYSIWYG [13] concatenates different frames into a single frame to expand the visibility area, enabling a broader perspective in the detection process. Another method, 3DVID [35], explores spatial–temporal correlations between multiple frame point clouds by using a Spatial Transformer Attention (STA) module to suppress the background noise and emphasize objects and a Temporal Transformer Attention (TTA) module to correlate the moving objects between frames.

## 3. Methods

**Figure 4:**
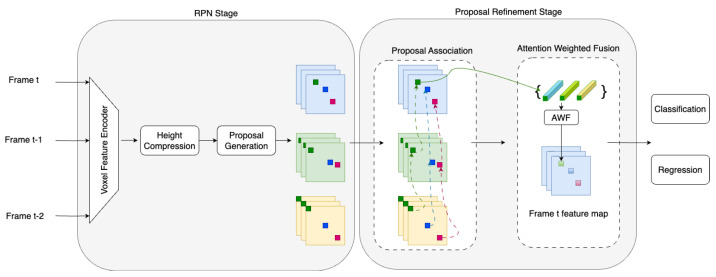
The structure of our approach. The yellow, green, and blue squares refer to the feature maps extracted from different frames, and the green, blue, and red blocks refer to the proposals in each frame. The proposal refinement stage contains two modules: proposal association and feature fusion.

The framework of the 3D object detection method based on multiple-frames fusion is shown in Figure 4. We use the LiDAR point cloud as the input and adopt the two-stage detection framework: region proposal network (RPN) and proposals refinement network. In the preprocessing stage, we use a voxel feature encoder (VFE) to encode the input point cloud data as voxels. In the RPN stage, we extract features from voxels and generate the prediction according to the anchor feature to obtain high-quality 3D proposals. Then, non-maximum suppression (NMS) is used to select candidate proposals in the proposal refinement stage. We associate and merge the features of 3D proposals from consecutive frames. Cosine similarity is used to associate proposals in consecutive frames, and the AWF module is used to adaptively adjust the features from matched proposals. Based on the fusion results, the bounding box classification and regression determine the object category, size, and location.

### 3.1. RPN Stage

Point cloud data form a disordered 3D point set. A point cloud is divided into 3D grids (voxels) in the preprocessing. Given an input point cloud with depth, height, and width of (D,H,W) and a predefined voxel size of $\left(v_{D},v_{H},v_{W}\right)$, the entire input point cloud will be divided into $\left(\frac{D}{V_{D}},\frac{H}{V_{H}},\frac{W}{V_{W}}\right)$ voxels along each coordinate axis. These voxels are then encoded to generate features and extract multi-scale 3D features. Subsequently, compression is applied to the z-axis to obtain a pseudo-2D feature map. In this process, the network only processes non-empty voxels to speed up the feature extraction process.

For the input voxelized point cloud (batch size, 3 + C, $\frac{D}{V_{D}},\frac{H}{V_{H}},\frac{W}{V_{W}}$), where C represents the number of additional information channels apart from the (x, y, z) information, usually include reflection intensity, time stamp, etc. The output features are the feature maps stacked on the z-axis direction. This network consists of two components: sparse convolution (spconv) and subconvolution (subconv).

Table 1 shows the structure of voxel feature encoding layers. spconv is composed of a 3 × 3 × 3 convolution with a stride of 2, followed by BatchNorm and ReLU activation. Spconv is used for performing downsampling. Subconv involves a 3 × 3 × 3 convolution with a stride of 1, followed by BatchNorm and ReLU activation. Subconv is used for feature extraction. Notably, only an eight-times downsampling is applied in the h and w directions. The last spconv layer has a stride of 2 in the z-axis direction. The final output features are represented by (batch size, channel, z, h, w), and a height compression operation is performed to stack the z-axis and channel dimensions, resulting in a pseudo-2D feature map which shape is (batch size, channel × z, h, w).

After obtaining the pseudo-2D feature map, two separate branches perform 2D convolutions with a kernel size of 3 × 3. Both conv2d and deconv operations are followed by BatchNorm and ReLU activation. In one branch, downsampling of 2 times is applied, followed by deconvolution to restore the feature map’s shape. The features from both branches are then concatenated to obtain multi-scale features. Finally, the multi-scale features are fed through the Conv2d layers for proposal prediction and regression. We use NMS to remove redundant proposals [15], and IOU = 0.45 as the threshold. The selected proposals will be kept for the Proposal Refinement Stage for refinement.

### 3.2. Proposal Refinement Stage

This stage aims to generate accurate 3D detection results from the candidate proposals through further optimization and regression. In this part, we find the proposals of the same object between consecutive frames through the feature association module. The AWF feature fusion module is then used to adaptively fuse the candidate frame features from different frames and send the fused results to the network for regression and classification.

#### 3.2.1. Feature Association Module

For proposal sets $P_{t},P_{t-1}$ are generated from the region proposal network from $F_{t},F_{t-1}$, separately, where $p_{i}=\left(x_{i}^{\prime },y_{i}^{\prime },z_{i}^{\prime },w_{i}^{\prime },l_{i}^{\prime },h_{i}^{\prime },\theta_{i}^{\prime }\right)\in P_{t},p_{j}=\left(x_{j}^{\prime },y_{j}^{\prime },z_{j}^{\prime },w_{j}^{\prime },l_{j}^{\prime },h_{j}^{\prime },\theta_{j}^{\prime }\right)\in P_{t-1}$. One approach for establishing associations between box proposals in consecutive frames is utilizing the nearest object center distance metric. The position offset of the object’s center point between the multiple frames is calculated as follows:

(1) $$d=\sqrt{{\left(x_{i}-x_{j}\right)}^{2}+{\left(y_{i}-y_{j}\right)}^{2}+{\left(z_{i}-z_{j}\right)}^{2}}<d_{th}$$

where ($x_{i}$, $y_{i}$, $z_{i}$) and ($x_{j}$, $y_{j}$, $z_{j}$) represents the proposal center in multiple frames, respectively. $d_{th}$ is a manually set threshold. When there is more than one proposal inside this threshold, the nearest proposal would be chosen to fuse.

We also consider using cosine metric distance as the correlation metric and calculate it using the potential features obtained from the network. Compared with Euclidean distance, cosine similarity is more sensitive to the pattern of two features, which is widely used in many applications [36,37]. That is,

(2) $$\begin{array}{c} \cos\left(\theta \right)=\frac{F_{t}\cdot F_{t-1}}{∥F_{t}∥∥F_{t-1}∥}=\frac{\sum_{k=1}^{n}F_{t_{k}}F_{t-1_{k}}}{\sqrt{\sum_{k=1}^{n}F_{t_{k}}^{2}}\sqrt{\sum_{k=1}^{n}F_{t-1_{k}}^{2}}} \end{array}$$

where $F_{t_{k}}$, and $F_{t-1_{k}}$ represent components of feature $F_{t}$ and $F_{t-1}$ respectively. $F_{t}$ and $F_{t-1}$ are consecutive frames. θ donates the angle between two features.

#### 3.2.2. Feature Fusion Module

The addition operation is a commonly used feature fusion method. That is,

(3) $$F_{t}^{\prime }=\sum_{k=1}^{n}\left(F_{t_{k}}+F_{t_{k-1}}\right)$$

where $F_{t_{k}}$, and $F_{t-1_{k}}$ represent components of feature $F_{t}$ and $F_{t-1}$ respectively. $F_{t}$ and $F_{t-1}$ are consecutive frames and + is the element-wise addition operation. However, the addition operation could be contextual unawareness [38]. Here, we use addition operation as a fusion method baseline and introduce an attention-weighted fusion (AWF) module to put weights into feature channels and fuse the features adaptively.

**Figure 5:**
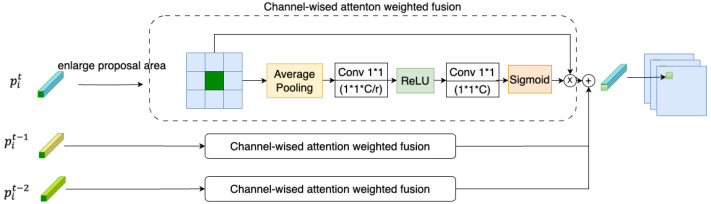
Attention-weighted fusion flowchart.

As shown in Figure 5, the input proposal is first enlarged to 3 × 3 on the feature map to include the surrounding areas for additional information. The module performs average pooling on the input feature map, which reduces the spatial dimensions to 1 × 1 while preserving the number of channels. The module applies a 1 × 1 convolution on the pooled tensor, which reduces the dimension of the channel. This is accomplished using a smaller number of output channels (C/r) compared to the input channels, which projects the feature into a lower-dimensional space and removes redundant information. The output tensor is passed through a ReLU function, which introduces nonlinearity into the feature representation. Following this, the module applies another 1 × 1 convolution, which expands the dimensionality of the feature back to the original number of channels. This convolution is followed by a sigmoid activation function, which scales the learned weights to the range [0, 1]. These weights represent the importance of each channel in the input feature map, with higher weights indicating more discriminative features. Finally, the input feature map is multiplied element-wise with the learned weights to obtain a weighted feature map. That is,

(4) $${\mathrm{Feature}}_{\mathrm{weighted}}={\mathrm{Channel}}_{\mathrm{attention}}⊗\mathrm{Feature}$$

(5) $${\mathrm{Channel}}_{\mathrm{attention}}=\mathrm{Sigmoid}(\mathrm{Conv}2(\mathrm{ReLU}(\mathrm{Conv}1({\mathrm{Avg}}_{\mathrm{Pooing}}(\mathrm{Feature})))))$$

## 4. Experimental Results

### 4.1. Dataset

NuScenes dataset uses Velodyne 32-beam LiDAR to collect the data. Compared with [39], which uses 64-beam LiDAR for data collection, the number of points in the nuScenes dataset is more sparse. There are 160,139 points per scene in the Kitti dataset, while only 24,966 points per scene in the nuScenes dataset. This indicates that the point density in nuScenes is five times lower than Kitti. Meanwhile, in the Kitti dataset, points within 70 m are annotated, while in the nuScenes dataset, points within 100 m are annotated. All these make the nuScenes dataset more challenging for users. The data collection of the nuScenes [29] is mainly carried out in Boston and Singapore, using one spinning LiDAR, five long-range RADAR sensors, and six cameras to collect data for these scenes. We use LiDAR data in our experiment. The dataset contains 1000 sequences, each of which lasts for about 20 s. Following the nuScenes pre-split policy, the dataset has 34,149 samples, divided into 28,130 training samples and 6019 validation samples. Ten types of objects are involved in this dataset: cars, trucks, construction vehicles ($c_{v}$), buses, trailers, motorcycles and bicycles, barriers, pedestrians, and traffic cones were considered to be small objects. The nuScenes dataset is a widely utilized multiple-frame dataset. Our research compared our approach and other existing methods on the nuScenes dataset.

The Kitti dataset is employed to assess our module performance. Only the ablation study is conducted on the Kitti dataset. As its detection set is a single frame dataset, we use Kitti tracking dataset sequence 5 for our ablation study to take advantage of multiple frame point clouds. In total, 200 samples are used for training and 97 are used for testing; only the vehicle category is included.

### 4.2. Implementation Details

We employ SECOND as our backbone network for the experiments. To verify the effectiveness of using multi-frame proposal features, we conducted the following experiments on the Kitti dataset: (a) single-frame input, (b) comparison of different feature association methods, (c) comparison of different feature fusion methods.

We follow the data augmentation strategy of [4,26], which includes random flipping along the x and y axes, random global rotation, and random global scaling. For the nuScenes dataset, the point cloud range is [−51.2, −51.2, −5.0, 51.2, 51.2, 3.0] meters, and the voxel size is [0.1, 0.1, 0.2] meters. The network outputs a point cloud feature map of size [512, 128, 128]. For the Kitti dataset, the point cloud range is [0, −40, −3, 70.4, 40, 1] meters, and the voxel size is [0.05, 0.05, 0.1] meters. The size of the output feature map is [512, 176, 200]. We use the same loss function as in SECOND [4]. We trained our model using two V100 GPUs. We train on the nuScenes dataset for 20 epochs with a batch size of four and the Kitti dataset for 60 epochs with a batch size of four. The Adam optimizer with an initial learning rate of 0.003 is utilized. We used mean Average Precision (mAP) as our evaluation metric to evaluate the performance.

### 4.3. Comparison with State-of-Art Results

Table 2 presents the mAP scores of detection methods on the nuScenes dataset. Pointpillars and 3DSSD are voxel-based and point-based single-frame methods, respectively. WYSIWYG is an extension of Pointpillar that utilizes concatenation to incorporate information from consecutive frames. The proposed method effectively utilizes features from proposal regions. It achieves an mAP score of 46.7, outperforming the state-of-the-art method 3DVID by 1.3%.

### 4.4. Component Studies

Table 3 illustrates the detection performance at different $d_{th}$ thresholds. $AP_{bev}$ refers to the average precision under bev view and $AP_{3d}$ refers to the average precision under 3D. Objects are divided into easy, moderate, and hard according to Kitti [39] standard, according to their occlusion level. For all the results, the higher the better. The bold indicates the best result. It is observed that using Euclidean distance as the correlation method is sensitive to threshold selection. This issue is more severe for outdoor datasets with multiple varying object speeds.

Table 4 compares different association methods when employing the add operation to fuse the features. The “No fusion” method represents our single-frame backbone network [4]. Add refers to the addition for feature fusion, EU refers to Euclidean distance for feature association, and cos refers to cosine similarity. We use the best EU threshold for this comparison, where dth = 5. In the addition method, the difference in accuracy between the EU and Cos-based methods is insignificant in the bird’s-eye-view (BEV) domain. However, the Cos-based method significantly improves 3D bounding box regression. The disadvantage of EU distance calculation is that it requires a manually set threshold. However, as discussed, this may wrongly associate nearby proposals. The performances of addition operation methods are superior to that of the no-fusion approach, indicating that utilizing features from proposal areas results in improved detection performance.

We propose the Attention-Weighted Fusion (AWF) module that enables adaptive weighting of different channels in proposal features, thereby suppressing irrelevant information and enhancing relevant features. By dynamically adjusting the feature weights, the AWF module effectively emphasizes useful information while attenuating the impact of less informative channels and improving feature fusion performance.

Table 5 presents the performance of different fusion methods when associating proposal features using cosine similarity. AWF refers to the Attention-Weighted Fusion module. For both BEV and 3D bounding box regression, Attention-Weighted Fusion outperforms the add operation by 2–4%. Notably, it significantly improves the detection accuracy of ‘hard’ objects, with an increase of 4.045% in BEV regression and 3.7877% in 3D regression. This observation highlights that the AWF module can effectively emphasize key features for improved object detection performance.

Table 6 presents the performance of different similarity methods when fusing proposal features using AWF. Multiheaded refers to multiheaded similarity. Combining cosine similarity and AWF outperforms other similarity methods for BEV and 3D bounding box regression. The reason for not using multiheaded similarity is because, when carrying out the proposal association, the feature that represents the proposal is [1, 1, 512]. As the 512 dimension is gained from height compression, one main reason to focus on the channel information is that it contains the height information of a proposal. Thus, this is not a complex pattern. The later AWF module helps pay attention to height patterns in the features.

**Figure 6:**
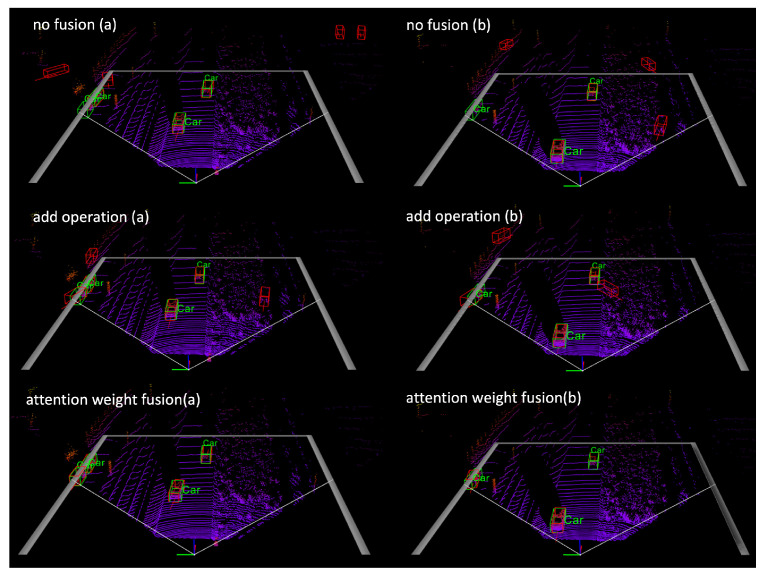
Ablation experiment. Multiple-frame detection result visualization with different fusion methods. Green boxes refer to the ground truth boxes, and red boxes are prediction results.

In Figure 6, we use a set of samples from the Kitti validation set as an example in ablation experiments. The output results are visualized to compare the differences between the models qualitatively. (a) and (b) refers to two multiple frames. Green boxes refer to the ground truth boxes, and red boxes are prediction results. The “no fusion” method refers to our backbone network [4], which serves as the baseline for comparison in this experiment. We compare the detection results of two fusion techniques, “add operation” and “attention weighted fusion”. The association in both cases is performed using the cosine similarity metric. In frame (a), the minimum number of object points on the left side of the point cloud is 42, while in frame (b), the minimum number of points representing objects is 64. As can be seen from the figure, there are difficulties in detecting side objects in the “no fusion” method without information from adjacent frames, and false positives appear due to interference from irrelevant points. Utilizing a feature-merging strategy that integrates features from multiple frames has been shown to be effective in detecting objects with sparse points. Based on our analyses, the proposed method can detect objects accurately by using the AWF module to assign weights adaptively. Additionally, the AWF module adds a small computational cost. The additional overhead is two 1 × 1 convolution layers for each frame computation. It takes 2.886 ms for AWF to fuse features for the current frame on GPU V100.

## 5. Conclusions

This paper proposes a feature fusion method for 3D object detection based on multiple-frame proposals. Our method adopts a cosine similarity metric to associate features and leverages an attention module to fuse proposal features, which is both lightweight and accurate. The experiments show the effectiveness of our components, and the result shows that our approach outperforms the concatenation method WYSIWYG by 11.2% and state-of-the-art 3DVID by 1.3% on the nuScenes dataset.

## Figures and Tables

**Table 1:** Structure of Voxel Feature Encoding.

Operation Name		Opration Parameters				Output Features
		Kernel Size	Stride	Padding	Channel	
conv input	Subconv	3 × 3 × 3	[1, 1, 1]	[1, 1, 1]	16	(41, 1600, 1408)
conv1	Subconv	3 × 3 × 3	[1, 1, 1]	[0, 0, 0]	16	(41, 1600, 1408)
conv2	Spconv	3 × 3 × 3	[2, 2, 2]	[1, 1, 1]	32	(21, 704, 800)
	Subconv	3 × 3 × 3	[1, 1, 1]	[0, 0, 0]	32	(21, 704, 800)
	Subconv	3 × 3 × 3	[1, 1, 1]	[0, 0, 0]	32	(21, 704, 800)
conv3	Spconv	3 × 3 × 3	[2, 2, 2]	[1, 1, 1]	64	(11, 400, 352)
	Subconv	3 × 3 × 3	[1, 1, 1]	[0, 0, 0]	64	(11, 400, 352)
	Subconv	3 × 3 × 3	[1, 1, 1]	[0, 0, 0]	64	(11, 400, 352)
conv4	Spconv	3 × 3 × 3	[2, 2, 2]	[1, 1, 1]	64	(5, 200, 176)
	Subconv	3 × 3 × 3	[1, 1, 1]	[0, 0, 0]	64	(5, 200, 176)
	Subconv	3 × 3 × 3	[1, 1, 1]	[0, 0, 0]	64	(5, 200, 176)
conv out	Spconv	3 × 3 × 3	[3, 1, 1]	[2, 1, 1]	128	(2, 200, 176)

On the output feature map column, we use our parameters on the Kitti dataset as an example.

**Table 2:** Performance comparison of 3D object detection on NuScenes dataset (%).

Method	mAP	Car	Ped	Bus	Barrier	TC	Truck	Motor	Trailer	Bicycle	CV
PointPillars [19]	30.5	68.4	59.7	28.2	38.9	30.8	23.0	23.4	27.4	1.1	4.1
WYSIWYG [13]	35.4	80	66.9	54.1	34.5	27.9	35.8	18.5	28.5	0	7.5
3DSSD [17]	42.7	81.2	70.2	61.4	48.0	31.1	47.2	36.0	30.4	8.6	12.6
DVID [35]	45.4	79.7	76.5	47.1	48.8	58.8	33.6	40.7	43	7.9	18.1
PFF	46.7	79.3	75.3	62.4	51.4	53.9	49.9	30.6	39.1	11	14.2

TC refers to traffic cone, and CV refers to construction vehicle.

**Table 3:** Component study: dth threshold (%).

	${\mathrm{AP}}_{\mathrm{bev}}$			${\mathrm{AP}}_{\mathrm{3d}}$		
Method	Easy	Moderate	Hard	Easy	Moderate	Hard
dth = 1	88.6772	83.2126	83.1767	81.6382	77.8125	76.0544
dth = 2	84.1826	85.392	85.3143	76.7546	74.0287	74.0257
dth = 3	88.2759	82.7352	82.6947	81.2487	74.069	74.0394
dth = 4	86.8243	82.7479	82.4786	81.2243	76.8276	76.6873
dth = 5	91.8821	86.2608	86.0856	81.3464	78.2803	77.9954
dth = 6	84.977	83.3424	81.5064	76.1668	74.1699	74.1548
dth = 7	90.3835	86.8521	86.6164	77.1007	76.2283	76.2663
dth = 8	85.7249	82.1796	82.1344	82.68	75.3728	75.1641
dth = 9	88.2979	87.4722	85.2619	79.6411	75.9922	73.9868

**Table 4:** Component study: different association methods (%).

	${\mathrm{AP}}_{\mathrm{bev}}$			${\mathrm{AP}}_{\mathrm{3d}}$		
Method	Easy	Moderate	Hard	Easy	Moderate	Hard
No Fusion	87.2933	81.0305	80.984	77.2405	72.5922	72.3705
Add + EU	91.8821	86.2608	86.0856	81.3464	78.2803	77.9954
Add + Cos	91.1244	85.04	84.9569	87.1227	80.3082	78.4103

**Table 5:** Component study: different fusion methods (%).

	${\mathrm{AP}}_{\mathrm{bev}}$			${\mathrm{AP}}_{\mathrm{3d}}$		
Method	Easy	Moderate	Hard	Easy	Moderate	Hard
Add + Cos	91.1244	85.04	84.9569	87.1227	80.3082	78.4103
AWF + Cos	94.3175	89.0463	89.0018	89.8324	82.2408	82.198

**Table 6:** Component study: different similarity methods (%).

	${\mathrm{AP}}_{\mathrm{bev}}$			${\mathrm{AP}}_{\mathrm{3d}}$		
Method	Easy	Moderate	Hard	Easy	Moderate	Hard
AWF + Multiheaded	91.4002	85.663	85.5672	87.7026	78.9306	78.8764
AWF + Cos	94.3175	89.0463	89.0018	89.8324	82.2408	82.198

## Data Availability

Publicly available datasets were analyzed in this study. These data can be found here accessed on 23 August 2013: https://www.cvlibs.net/datasets/kitti and accessed on 31 March 2019 https://www.nuscenes.org/nuscenes.
